# Bortezomib-Induced Painful Peripheral Neuropathy: An Electrophysiological, Behavioral, Morphological and Mechanistic Study in the Mouse

**DOI:** 10.1371/journal.pone.0072995

**Published:** 2013-09-12

**Authors:** Valentina A. Carozzi, Cynthia L. Renn, Michela Bardini, Grazia Fazio, Alessia Chiorazzi, Cristina Meregalli, Norberto Oggioni, Kathleen Shanks, Marina Quartu, Maria Pina Serra, Barbara Sala, Guido Cavaletti, Susan G. Dorsey

**Affiliations:** 1 Department of Surgery and Translational Medicine, University of Milan Bicocca, Monza, Italy; 2 School of Nursing, Center for Pain Studies, University of Maryland, Baltimore, Maryland, United States of America; 3 “M. Tettamanti” Research Center, Department of Health Sciences, University of Milan Bicocca, Monza, Italy; 4 Department of Biomedical Sciences, Section of Cytomorphology, University of Cagliari, Monserrato, Italy; University of Arizona, United States of America

## Abstract

Bortezomib is the first proteasome inhibitor with significant antineoplastic activity for the treatment of relapsed/refractory multiple myeloma as well as other hematological and solid neoplasms. Peripheral neurological complications manifesting with paresthesias, burning sensations, dysesthesias, numbness, sensory loss, reduced proprioception and vibratory sensitivity are among the major limiting side effects associated with bortezomib therapy. Although bortezomib-induced painful peripheral neuropathy is clinically easy to diagnose and reliable models are available, its pathophysiology remains partly unclear.

In this study we used well-characterized immune-competent and immune-compromised mouse models of bortezomib-induced painful peripheral neuropathy. To characterize the drug-induced pathological changes in the peripheral nervous system, we examined the involvement of spinal cord neuronal function in the development of neuropathic pain and investigated the relevance of the immune response in painful peripheral neuropathy induced by bortezomib.

We found that bortezomib treatment induced morphological changes in the spinal cord, dorsal roots, dorsal root ganglia (DRG) and peripheral nerves. Neurophysiological abnormalities and specific functional alterations in Aδ and C fibers were also observed in peripheral nerve fibers. Mice developed mechanical allodynia and functional abnormalities of wide dynamic range neurons in the dorsal horn of spinal cord. Bortezomib induced increased expression of the neuronal stress marker activating transcription factor-3 in most DRG. Moreover, the immunodeficient animals treated with bortezomib developed a painful peripheral neuropathy with the same features observed in the immunocompetent mice.

In conclusion, this study extends the knowledge of the sites of damage induced in the nervous system by bortezomib administration. Moreover, a selective functional vulnerability of peripheral nerve fiber subpopulations was found as well as a change in the electrical activity of wide dynamic range neurons of dorsal horn of spinal cord. Finally, the immune response is not a key factor in the development of morphological and functional damage induced by bortezomib in the peripheral nervous system.

## Introduction

Bortezomib is the first proteasome inhibitor with significant antineoplastic activity for the treatment of relapsed/refractory multiple myeloma (MM) [1,2,3] as well as a variety of other hematological and solid neoplasms [4,5]. It acts through high-affinity and specific binding of its boron atom to the catalytic site of the 26S proteasome [6]. A variety of mechanisms are involved in the anti-proliferative effect of bortezomib, including reversible inhibition of the proteasome and NF-κB signaling pathway, which inhibits anti-apoptotic factors and permits the activation of programmed death in cancer cells [7,8]. Peripheral neurological complications are among the major side effects associated with bortezomib therapy particularly if given intravenously [9] and they severely affect normal activities of daily living in MM patients. Bortezomib-induced peripheral neuropathy (PN) is characterized by paresthesias, burning sensations, dysesthesias, numbness, sensory loss, reduced proprioception and vibratory sensation that presents in a stocking-and-glove distribution. Deep tendon reflexes are also reduced [10,11,12,13], while motor impairment is generally only subclinical above all when patients had a pre-existing neuropathy. Reduced autonomic innervation in the skin of bortezomib-treated patients has also been reported [14]. The most clinically relevant bortezomib-induced adverse effect is neuropathic pain, evident as abnormal touch detection (mechanical allodynia) and reduced thermal thresholds that usually do not subside between courses of therapy [12]. Although bortezomib-induced painful PN is easy to diagnose, its pathophysiology remains unclear. Peripheral neuropathic pain is attributed to plastic changes that affect either the primary afferent fibers or their synapses in the central nervous system (CNS). These changes include peripheral/central sensitization [15,16] and alterations in the function of CNS centers involved in the processing of nociceptive information [17,18]. If and how bortezomib, which does not cross the blood brain barrier, causes alterations in the central part of sensory pathways remains to be elucidated.

In studies of rat and mouse models, chronic treatment with bortezomib induces a significant and dose-dependent reduction of nerve conduction velocity (NCV), resulting from mild to moderate pathological changes that involve both myelinated and unmyelinated peripheral nerve fibers. Moreover, intracytoplasmic vacuolization of satellite cells and sensory neurons, due to mitochondrial and endoplasmic reticulum damage, was observed in dorsal root ganglia (DRG) [19,20,21]. However, the molecular alterations that occur in the DRG and peripheral nerves of bortezomib-treated animals remain unclear. At the behavioral level, bortezomib-treated animals develop mechanical and thermal allodynia [20,22] and sensory-motor function changes [22], but not thermal hyperalgesia [20].

Various mechanisms involved in the development of bortezomib-induced painful PN have been explored, such as oxidative stress [23], mitochondrial damage [24] and altered glutamate signaling [25]. While the role of the immune response in the development of bortezomib-induced painful PN remains unclear, inflammation has been described as a key event in the development of neuropathic pain induced by other chemotherapy drugs [26,27,28]. In fact, it is accepted that neuropathic pain results from damage or inflammation of the nervous system inducing painful conditions and hypersensitivity phenomena described as allodynia [29]. Furthermore, immune modulation therapy has been proposed for use in the management of bortezomib-induced PN [30].

In this study, we used immune-competent and immune-compromised mouse models of bortezomib-induced PN to 1) determine the involvement of spinal cord neuronal function during painful PN, 2) further characterize the pathological changes in the DRG and 3) investigate the relevance of the immune response in the development of painful PN induced by chronic bortezomib administration.

## Methods

### 1: Animals

Young adult female BALB/c mice (~20 g, Harlan, San Pietro al Natisone, Italy or Harlan Laboratory, Frederick, MD, USA) were used for the study. Mice, that underwent X-Ray irradiation to become immune-compromised, were housed in a dedicated room with Individually Ventilated Cages (IVC, Tecniplast spa, Varese, Italy). All mice were housed on a 12: 12 h light: dark cycle with food and water available *ad libitum.*

### 2: Ethics Statements

All the procedures on animals were in compliance with international policies (EEC Council Directive 86/609, OJ L 358, 1, Dec.12, 1987; Guide for the Care and Use of Laboratory Animals, US National Research Council, 8th ed., 2011). The International Association for the Study of Pain (IASP) guidelines for the investigation of pain in animals were followed [31]. The Institutional Animal Care and Use Committee of the University of Maryland School of Medicine and the Ethics Committee for Animal Studies of the University of Milan-Bicocca approved all the experiments (permit numbers: 0710003, 2d_CE_16/01/2012, respectively). All the mice were euthanized four days after the end of the drug treatment.

### 3: Anesthesia

For peripheral blood (PB) and femoral bone marrow (BM) collection, neurophysiology and electrophysiological recordings, anesthesia was induced in a chamber with 3% isoflurane carried in oxygen followed by 1-1.5% isoflurane by nose cone for maintenance throughout the procedures, which was adequate to suppress the corneal blink response and any withdrawal response to a noxious stimulus. For the Neurometer test, anesthesia was induced in a chamber with 3% isoflurane carried in oxygen followed by 0.75-1% isoflurane by nose cone for maintenance throughout the procedure, which was adequate to keep the mouse restrained but still allowed a hind paw withdrawal response to the stimuli. Additionally, prior to the laminectomy surgery for the spinal cord electrophysiological recordings, mice were intraperitoneally injected with Pentobarbital 40 mg/kg. To minimize isoflurane-induced hypothermia, the body temperature was maintained at ~37°C using a heating pad (Homeothermic System, Harvard Apparatus, Holliston, MA).

### 4: Drug

Bortezomib (LC Laboratories, Woburn, MA) was prepared immediately before each administration and dissolved in a vehicle solution composed of absolute ethanol/tween80/saline (5%/5%/90%). A 10 ml/kg volume was administered intravenously in the caudal vein. Bortezomib at 0.8 mg/kg or a dose-equivalent volume of vehicle solution was injected twice/weekly for 4 weeks. The treatment schedule was chosen on the basis of previously reported data [21].

### 5: Experimental Design

For each assay, the investigator was blinded as to the experimental condition of mice. The experimental plan (summarized in Table **1**) was divided into two parts: the first part (**experiment 1**) was carried out to fully characterize the bortezomib-induced painful PN in BALB/c mice; the second part (**experiment 2**) aimed at investigating the role of the immune response in the development of painful PN.

**Table 1:** Summary of the experimental plan.

**EXPERIMENT 1**	**CONDITIONS**	**#**	**ANALYSIS**
CLUSTER 1	NAÏVE, VEH_TR,_ BTZ_TR_	8	ELECTROPHYSIOLOGY IN S.C
CLUSTER 2	NAÏVE, VEH_TR,_ BTZ_TR_	8	BEHAVIORAL TESTS, NEUROMETER ANALYSIS
CLUSTER 3	NAÏVE, VEH_TR,_ BTZ_TR_	8	NEUROPHYSIOLOGY, MORPHOLOGY, IMMUNOHISTOCHEMISTRY FOR ATF3
**EXPERIMENT 2**	**CONDITIONS**	**#**	**ANALYSIS**
CLUSTER 1	NAÏVE, VEH_TR,_ BTZ_TR_	8	FLOW CYTOMETER, MORPHOLOGY, BEHAVIORAL TEST, NEUROPHYSIOLOGY

The experimental plan was composed of two experiments. In experiment 1, three clusters of mice were employed. Within each cluster, the animals were randomized into 3 groups, one injected with bortezomib (BTZ _TR_), one with vehicle (VEH _TR_) and one left untreated (NAÏVE). The first cluster of animals was used for the electrophysiological analysis in the dorsal horn of the spinal cord, the second for the behavioral tests and Neurometer analysis and the third for the neurophysiological, histopathological and immunohistochemical analyses.

In experiment 2, one cluster of animals was randomized into three groups: one group of mice was exposed to the X-Ray immunosuppressive irradiation (X-Ray_TR_), one group was exposed to X-Ray immunosuppressive irradiation and then injected with bortezomib (X-Ray_TR_ + BTZ_TR_) and a group was left untreated (NAÏVE). Flow cytometer, histopathological, behavioral and neurophysiological analyses were performed.

TR = treated; # = number of mice/group; VEH = vehicle; BTZ = bortezomib; S.C = spinal cord.

In experiment **1**, all of the studies used the same schedule of bortezomib administration in 3 clusters of 24 mice each. The first cluster of mice was used for spinal cord electrophysiological assessments. The second cluster was used for the behavioral and Neurometer tests. The third cluster was used for the peripheral nerve neurophysiological measures, the morphological observations of DRG, spinal cord, sciatic and caudal nerve, dorsal and ventral roots and for the immunohistochemical localization of the neuronal stress marker Activating Transcription Factor (ATF) -3. In each experimental paradigm mice were randomly assigned to 3 experimental groups of 8 mice each: one group was treated with bortezomib 0.8 mg/kg/i.v. twice weekly for 4 weeks, one group was treated with a dose-equivalent volume of vehicle i.v. twice weekly for 4 weeks and a group of naïve mice was left untreated.

**Figure 1:**
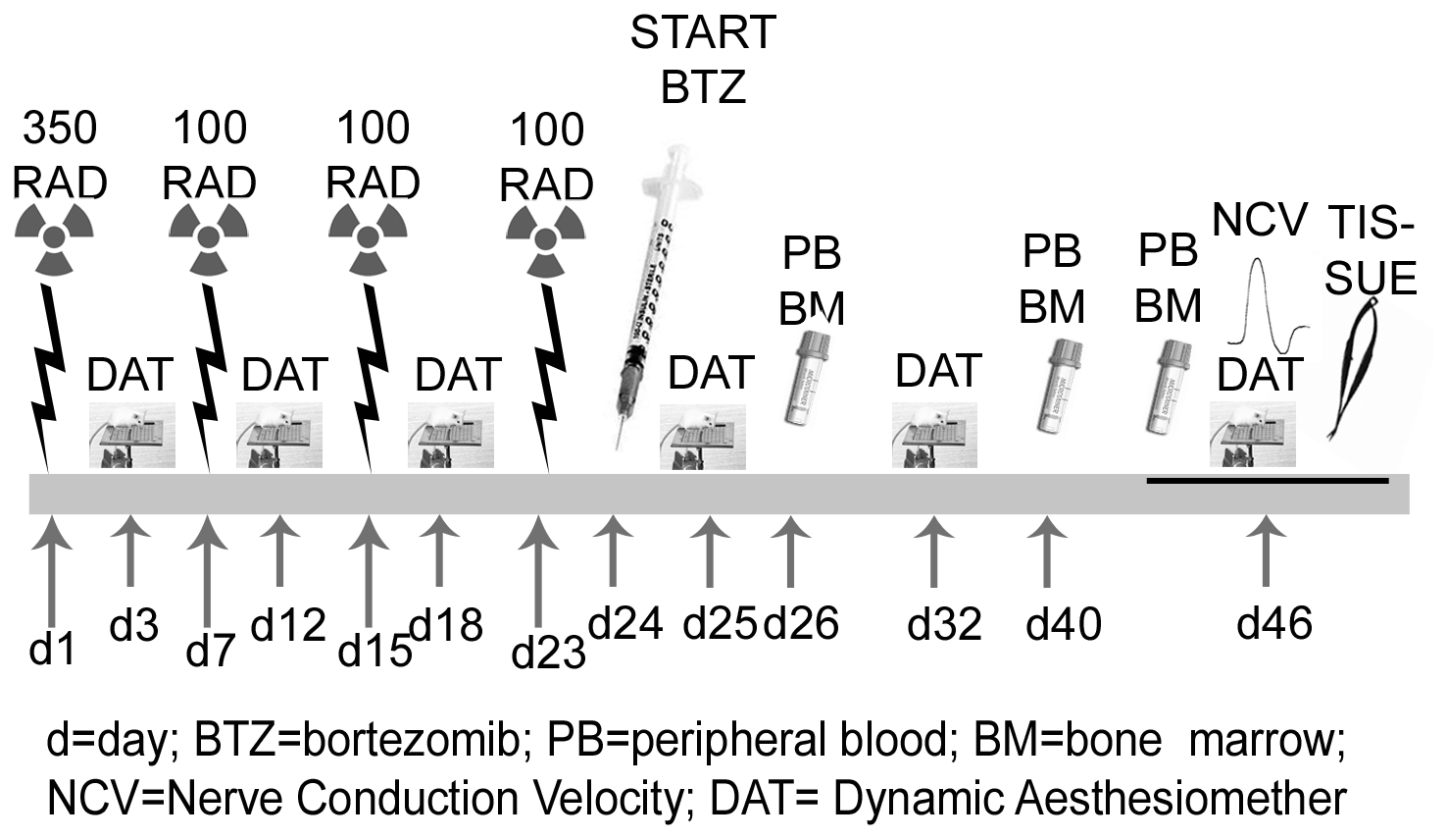
Flow chart of experiment 2. In experiment 2, animals were exposed to X-Ray irradiation on day 1, 7, 15 and 23. Twenty-four hours after the last irradiation, animals started the 4-week period of bortezomib chemotherapy. The Dynamic Aesthesiometer Test was performed on day 3, 12, 18, 25, 32, 46 and the cytofluorimetric analysis of PB and BM CD45 positive cells on days 26, 40 and 46. On day 46 animals underwent the neurophysiologic analysis and, once euthanized, the sample collection for the neuropathological analysis.

In experiment **2** (time-course is summarized in Figure **1**), mice were randomly assigned to 3 groups (n=8/group) and all housed under the conditions previously described for immunodeficient mice. One group of mice underwent X-Ray irradiation to deplete the immune cell-mediated response and one group of mice was exposed to the X-Ray irradiation and then treated with bortezomib according to the same schedule of **experiment 1**. A third group of naïve mice was left untreated. Flow cytometer analysis of the PB and femoral BM was performed on days 26, 40 and 46 to assess the number of CD45-positive cells, a surface marker of both B and T cell-derived subpopulations in mice. In both experiments the development and severity of PN was assessed by neurophysiological analysis of the peripheral nerves, morphological analysis of DRG and peripheral nerves and pain perception threshold using a behavioral test for mechanical sensitivity.

### 6: X-Ray Irradiation

X-Ray irradiation was performed under sterile conditions using a RADGIL X-Ray treatment unit (Gilardoni, Mandello del Lario, Italy). Mice of **experiment 2** were placed in a Rad Disk box and exposed to a 350 RAD (7.4 min) sub-lethal dose of radiation exposure on day 1 followed by three 100 RAD (2.1 min) maintenance exposures on days 7, 15 and 23 (Figure **1**). In the 2 weeks after irradiation, mice were treated with Ciprofloxacin (100 mg/L) dissolved in their drinking water to prevent the development of opportunistic bacterial infections as a consequence of the irradiation-induced immunosuppression.

### 7: General toxicity

Mice were examined for sickness symptoms due to drug treatment and irradiation-induced immunosuppression. Changes in their appearance (decreased grooming, dishevelled fur, piloerection, exaggerated kyphosis), behavior (decreased nesting) and activity (decreased exploring) was monitored daily. The body weight was recorded twice weekly to assess the general toxicity of the treatments and to calculate the weight-based bortezomib dose.

### 8: Bone marrow and peripheral blood collection

The femoral BM and PB were collected under deep anesthesia from 3 mice/group on days 26, 40 and 46 of **experiment 2** (Fig.**1**). The same mice were not analyzed more than once to avoid repeated invasive procedures. The BM was collected by femoral aspiration using a 300µl micro-fine insulin-syringe with a 30-gauge needle (BD Biosciences, USA) and analyzed to determine the number of CD45-positive cells using a flow cytometer. A PB sample was drawn from the sub-mandibular plexus by a single puncture using an 18-gauge needle and analyzed to determine the white blood cell (WBC) count. After the BM and PB drawing procedures, the mice did not show any signs of distress.

At the completion of all of the experiments, the mice were euthanized and the BM cells processed by crushing the femurs using a mortar and pestle. The cells were phenotypically analyzed as described below (section 8).

### 9: White blood cells count and flow cytometer analysis

PB and BM samples were collected as described above in **experiment 2**. Cellularity was assessed using a Coulter AcT diff hematology analyzer instrument (Beckman Coulter, Cassina De’ Pecchi, Italy). Red blood cells were lysed with ammonium chloride solution (Voden Medical Instruments, Peschiera Borromeo, Italy) to purify leukocytes. Cells were stained with the following mouse antibodies: CD45.2-PE or –PerCPCy5.5 (pan-leukocyte antigen), CD19-PE (B-cell antigen), CD3-APC (T-cell antigen), Gr1-APC (myeloid antigen), NK1.1-PE (natural killer cell antigen) (all from Bioscience inc. San Diego CA, USA); and subsequently analyzed by flow cytometry using a FACS Calibur and Cell Quest Pro software (BD Biosciences, Buccinasco (MI), Italy).

### 10: Neurophysiological analysis of the peripheral nerves

Two days after the last bortezomib administration, caudal and digital NCVs and action potential amplitudes were measured using an electromyography instrument (Myto2 ABN Neuro, Firenze, Italy) as previously described [21]. Briefly, caudal NCV was measured by placing two proximal recording needle electrodes on the tail and two stimulating needle electrodes 3.5 cm distal to the recording electrodes. The digital NCV was measured in the hind paw by placing the recording electrodes near the ankle and the stimulating electrodes in the fourth toe. Both the caudal and digital NCVs were calculated as a ratio (m/sec) of the distance (cm) between stimulating and recording electrodes and the time latency (sec) from the stimulus artifact to the onset of the elicited action potential. Serial stimulations with different amplitudes (3-30 mA) were performed to achieve the maximal action potential feedback. Ten responses per stimulation amplitude were averaged for each recording.

### 11: Behavioral tests

#### 11.1: Mechanical stimulation: Dynamic Aesthesiometer test

The nocifensive behavior of paw withdrawal from a mechanical stimulus was used to assess the development of mechanical allodynia in experiment **1** and **2**. The Dynamic Aesthesiometer test (model 37450, Ugo Basile Biological Instruments, Comerio, Italy), which generates a linearly increasing mechanical force [32], was performed weekly from baseline through the end of bortezomib administration. Before performing the test at baseline, the mice were acclimated to the instrument for 30 min/day on two consecutive days. Testing was conducted on the third day. On each day of testing, the mice were placed in a Plexiglas chamber (28 x 40 x 35-cm) on a wire mesh platform for 30 min to acclimate followed by testing. After the acclimation period, a servo-controlled mechanical stimulator with a blunt metallic filament (0.5 mm diameter) was positioned under plantar surface of the hind paw and activated to exert a progressively increasing punctate pressure with a gram force ramp of 1 g/sec. When a clear hind paw withdrawal occurred, the stimulus was automatically stopped and the gram force of the pressure being applied at the time of withdrawal was recorded as the mechanical nociceptive threshold index. The mechanical threshold was assessed alternately on each hind paw every 2 min for three trials to obtain a mean value of the maximal pressure (expressed in grams) tolerated by the mice. If a paw movement subsequent to the onset of the stimulus appeared to be associated with grooming or locomotion, the stimulus was stopped by the investigator and repeated after a delay of 1 min. To prevent tissue damage, an upper limit cut-off of 15 g was set, after which the mechanical stimulus was automatically terminated.

#### 11.2: Thermal stimulation: Hot and Cold Plate Tests

In experiment **1**, an incremental hot/cold plate (PE34; IITC Life Sciences, Woodland Hills, CA) with a starting temperature of 30°C and the hot and cold ramps, set at the maximum rate of 10 °C/min, was used to induce the nocifensive behaviors of licking a hind paw and jumping to identify the thresholds for noxious heat and cold, respectively. The mice were tested once per week as previously described [33]. Briefly, each mouse was allowed to acclimate for 30-60 seconds in a Plexiglas cylinder on the 30°C metal plate prior to the onset of the stimulus trial. The temperature of the plate at the time when the licking (hot) or the jumping (cold) occurred was recorded as the outcome measure. Automatic cut-off temperatures of 0°C (cold stimulus) and 50°C (hot stimulus) were used to avoid tissue injury.

### 12: Electrophysiological analysis in the spinal cord

Electrophysiological analysis in the spinal cord was performed at the end of the treatment period in **experiment 1** to measure the electrical activity of wide dynamic range (WDR) neurons in the spinal dorsal horn. The surgical procedures and the electrophysiological recordings were carried out as previously described by Renn [33]. Briefly, a laminectomy was performed to expose the lumbar enlargement of the spinal cord. A fine (<1.0µm) high impedance tungsten microelectrode tip (8 Meg, Frederick Haer Co, Brunswick, ME) was inserted vertically to a depth of 400-650 µm from the dorsal surface of the spinal cord (Model 660 micropositioner, David Kopf Instruments, Tujunga, CA). Mechanical stimuli of varying intensities were applied to the plantar surface of the hind paw ipsilateral to the recording electrode using different tools (00 sable hair paint brush for 10-sec. and von Frey filaments 0.4g/1g/4g for 2-sec. each). The extracellular potentials were recorded, amplified and filtered using standard electrophysiological techniques. Neuronal activity was discriminated, sorted and analysed by principal components analysis offline using SciWorks (v7.0, Datawave Technologies, Berthoud, CO) and the stimulus-evoked neuronal activity was quantified by calculating the number of spikes/sec during the stimulation.

### 13: Current perception threshold measurements

For the current perception threshold (CPT) measurement in the footpad, the mice of **experiment 1** were placed in a restraint jacket and suspended from a frame 3 inches above the bench surface with the paws dangling freely [34]. Anesthesia was maintained by nose cone and titrated between 0.75-1% to allow a withdrawal response of the hind paw to occur without the mouse struggling to escape from the restraint. The stimulus electrode was applied to the plantar surface of the left hind paw and the ground electrode was applied to the left ankle. Using the Neurometer (Neurotron Inc., Baltimore, MD), sine-wave transcutaneous electrical stimuli were applied at three frequencies (2000Hz, 250Hz, and 5Hz) with the current increasing stepwise until a nocifensive response (paw withdrawal or flick) was observed. When a response was elicited, the stimulus was stopped and the amount of current (µA) delivered at the time of the response was recorded [35]. A nocifensive response was defined as a brisk withdrawal or flicking of the hind paw. The hind paw was tested three times at each frequency and the current perception threshold was the average of those three trials.

### 14: Histopathology

A 1-cm segment of lumbar spinal cord, sciatic nerves, caudal nerves, L4-L5 DRG, L4-L5 ventral and dorsal roots in **experiment 1** and sciatic and caudal nerves, DRG and 1-cm segment of lumbar spinal cord in **experiment 2** were harvested and processed according to previously reported protocols for the morphological analysis [21,36,37]. Briefly, tissues were fixed for 3 hours at room temperature in paraformaldehyde 4%/glutaraldehyde 2% (spinal cord and DRG) or in glutaraldehyde 3% (dorsal and ventral roots and peripheral nerves), post-fixed in OsO_4_ and epoxy resin embedded. Morphological analysis was carried out on 1 µm-thick semi-thin sections stained with toluidine blue. At least two tissue blocks for each animal were sectioned and then examined with a Nikon Eclipse E200 light microscope (Nikon, Firenze, Italy).

### 15: Immunohistochemistry for ATF3

From each DRG specimen harvested at the end of the **experiment 1**, 10 µm-thick cryostat sections were thoroughly washed in PBS (0.01 M, pH 7.4) at room temperature (RT), incubated in 3% H_2_O_2_ and then in 5% normal goat serum (NGS, Chemicon, Darmstadt, Germany) in PBS for 30 minutes. Following several rinses in PBS, the sections were incubated overnight at 4°C with rabbit anti-mouse ATF3 antibody (Santa Cruz Biotechnology, Dallas, TX), diluted 1:50 in PBS containing 1% NGS+0.2% Triton X-100. The following day the tissue was incubated in peroxidase linked-anti-rabbit antiserum (PerkinElmer, Waltham, MA), diluted 1:200 in PBS containing 1% NGS, for 1 h at RT. Both primary and secondary antibody optimal concentrations were determined in a preliminary set of experiments. The cellular immunoreactivity for ATF3 was visualized by the application of 3'-3'-diaminobenzidinewith 3% H_2_O_2_. The slices were then dehydrated, counterstained with toluidine blue staining, mounted with DPX mountant (BHD Laboratories, Poole, England) and observed with a microscope equipped with a digital acquisition system (Nikon, Firenze, Italy).

### 16: Statistical analysis

The differences between the groups within each experiment in CD45-positive cells content, body weight, electrophysiological and neurophysiological analysis, aesthesiometer tests, behavioral tests and Neurometer measurements were statistically analysed using the analysis of variance (One-way ANOVA, Tukey–Kramer post hoc-test). Statistical analysis was performed using GraphPad 3.0 software (GraphPad Software, La Jolla, CA). For all analyses, the *a priori* significance level was set at p<0.05.

## Results

### Experiment 1

#### 1: General appearance and assessment of body weight changes

**Figure 2:**
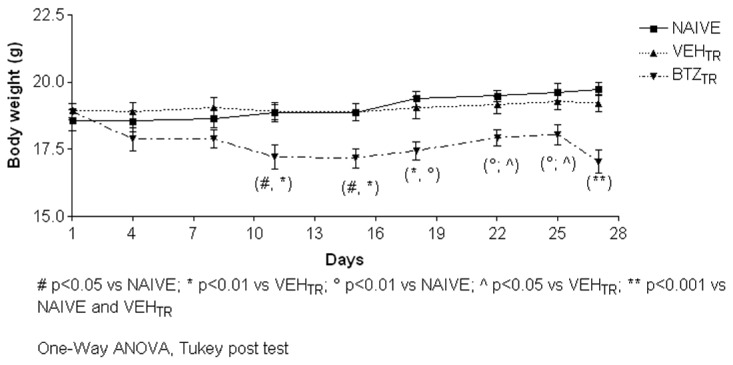
Body weight, experiment 1. Body weights were measured twice a week to monitor bortezomib general toxicity. Ten days after the first injection through the end of the experimental period, bortezomib caused a significant decrease in the body weight of drug-treated mice compared to the naïve and to the vehicle treated ones.

The bortezomib treatment was fairly well tolerated by the mice. No mice died or were euthanized prematurely during the experimental period and the majority of the bortezomib-treated mice continued to explore their environment, feed and groom. However, approximately 30% of the bortezomib-treated mice developed kyphosis, piloerection and pale skin. The body weight measurements showed that the bortezomib-treated mice started to lose weight after the first injection, which reached statistical significance after the fourth dose of the drug compared to naïve and vehicle-treated mice and persisted through the 28-day study period (Figure **2**; day 11 and 15, p<0.05 vs. naïve and p<0.01 vs. vehicle; day 18, p<0.01 vs. naïve and vehicle; day 22 and 25, p<0.01 vs. naïve and p<0,05 vs. vehicle; day 27, p<0.001 vs. naïve and vehicle).

#### 2: Neurophysiological assessments in the peripheral nerves

**Figure 3:**
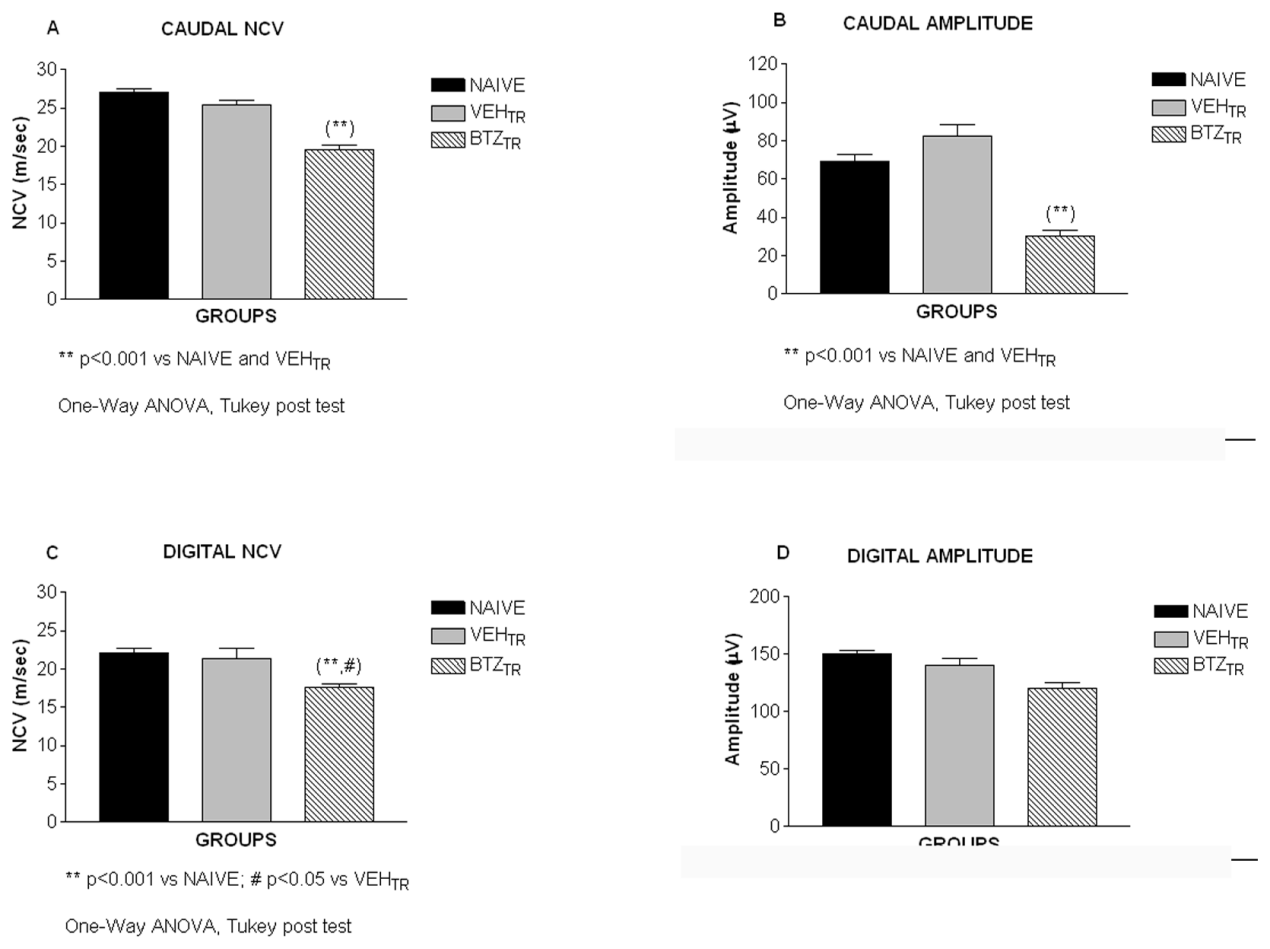
Neurophysiology, experiment 1. Nerve Conduction Velocity (NCV) and action potential amplitudes were tested with an electromyographic apparatus in the caudal and digital nerves two days after the last bortezomib administration. Bortezomib induced a significant decrease in the caudal (A) and digital (C) NCV compared to the naïve and vehicle-treated mice. Bortezomib induced a statistically significant reduction in the caudal action potential amplitude (B). There was no difference between naïve/vehicle and bortezomib-treated animals in the digital action potential amplitude (D).

Recordings of the NCV and neuronal action potential amplitude were performed on the digital and caudal nerves as a measure of the sensory and sensory/motor neurophysiological functionality, respectively. As reported in previous studies [21] Carozzi et al., 2010a), treatment with bortezomib induced a reduction of the caudal NCV (Figure **3A**; p<0.01 vs. naïve and vehicle-treated mice) and amplitude (Figure **3B**; p<0.05 vs. naïve and p<0.01 vs. vehicle-treated mice) and of the digital NCV (Figure **3C**; p<0.05 vs. naïve and vehicle-treated mice), but not of the digital amplitude (Figure **3D**; p>0.05vs. naïve and vehicle-treated mice).

#### 3: Assessments of mechanical and thermal thresholds

**Figure 4:**
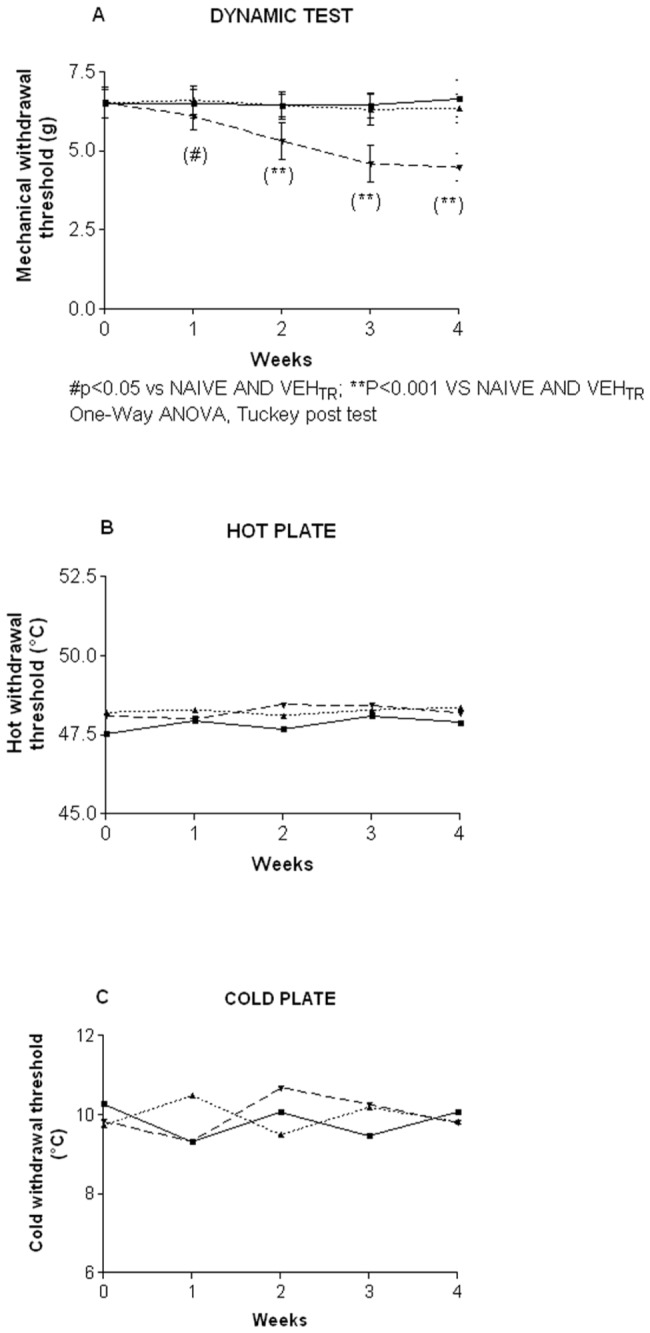
Mechanical and thermal thresholds, experiment 1. The mice were tested weekly using the Dynamic Aesthesiometer Test and the incremental hot/cold plate. Bortezomib induced a significant decrease in the mechanical threshold to induce a paw withdrawal at one week, which persisted through week 4 (A). There was no difference between naïve, vehicle and bortezomib-treated mice in the response threshold to a heat (B) and cold (D) stimulus.

The mice were tested at baseline, i.e. before starting drug treatment, and then weekly for 4 weeks to assess the nocifensive response to mechanical and thermal stimuli. The development of mechanical allodynia was determined using the Dynamic Aesthesiometer Test and was defined as a decrease in the paw withdrawal threshold compared to the vehicle and naive groups. No significant difference in withdrawal threshold was found between the groups at baseline (Figure **4A**). However, the bortezomib-treated mice had a significant decrease in mechanical threshold compared to the naïve and vehicle-treated group starting after 1 week of drug treatment and persisting through the fourth week (Figure **4A**; p<0.05 at week 1 and p<0.001 vs. naïve and vehicle-treated mice from week 2 to week 4). There was no statistically significant difference in the hot (Figure **4B**) or cold (Figure **4C**) response thresholds between the groups of mice at any time point after bortezomib treatment.

#### 4: Assessments of spinal cord neuronal electrical activity

**Figure 5:**
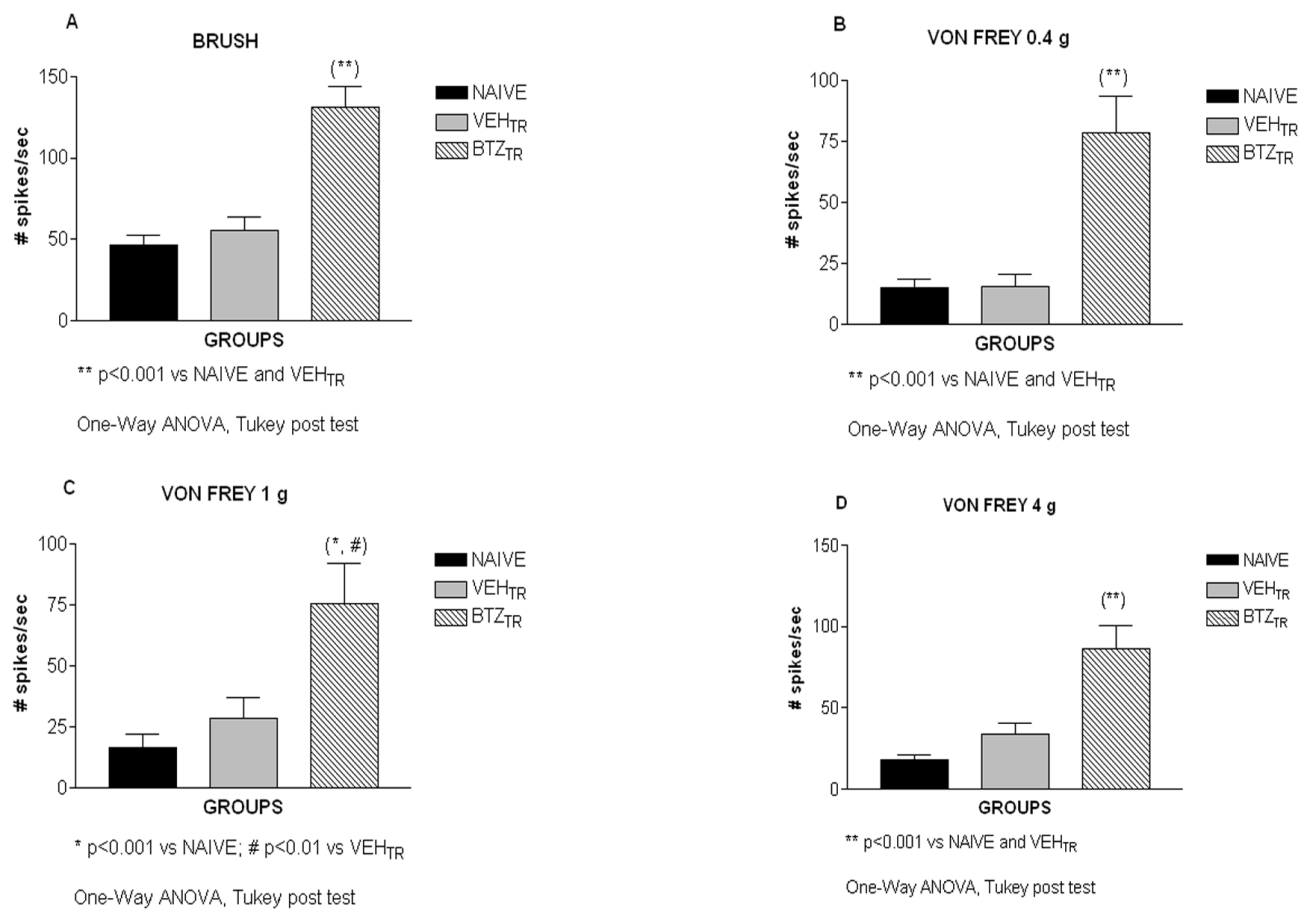
Electrophysiology of the spinal cord, experiment 1. Bortezomib increased the activity of wide dynamic range neurons in the spinal dorsal horn. Mice were tested for neuronal excitability during stimulation of the left hind paw with a brush and von Frey filaments (0.4, 1 and 4 grams). Bortezomib induced an increase of the number of spikes per second (spikes/s) compared to naïve and vehicle-treated mice in response to innocuous stimulation with a brush (A) and 0.4g filament (B), to moderate stimulation with the 1 g filament (C) and noxious stimulation with the 4 g (D) filament.

After determining that bortezomib induced the development of painful PN characterized by impaired neurophysiological function of peripheral nerves and by altered nocifensive behavioral responses to mechanical stimuli (allodynia), we investigated whether bortezomib treatment also induced changes in the activity of WDR neurons in the spinal cord dorsal horn. The electrical activity of 68 deep WDR neurons in the lumbar enlargement of the spinal dorsal horn (23 neurons from naive mice, 26 neurons from vehicle-treated mice and 19 neurons from bortezomib-treated mice) was measured. Extracellular electrophysiological recording demonstrated that the number of spikes per second was significantly higher in bortezomib-treated mice compared to naïve and vehicle-treated mice in response to innocuous (brush, 0.4g Von Frey filament), moderate (1 g von Frey filament) and noxious (4.0g von Frey filament) stimulation of the hind paw ipsilateral to the spinal recording site (Fig. 5 A, B C, D; brush, p<0.001 vs. naïve and vehicle-treated mice; von Frey filament. 0.4 g, p<0.001 vs. naïve and vehicle-treated mice; von Frey filament 1 g, p<0.001 vs. naïve and p<0.01 vs. vehicle-treated mice; von Frey filament. 4 g, p<0.001 vs. naïve and vehicle-treated mice).

#### 5: Assessment of different sensory fiber function

**Figure 6:**
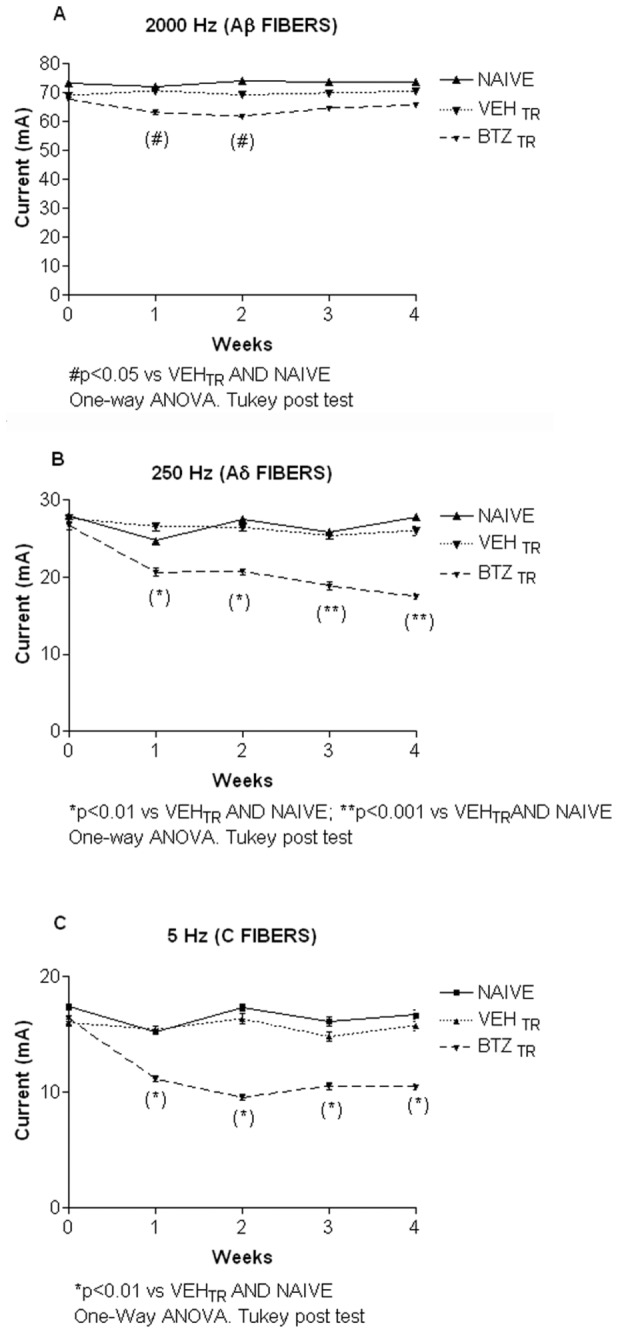
Neurometer, experiment 1. Bortezomib decreased the current perception threshold. The mice were treated with bortezomib or vehicle and tested weekly using the Neurometer to determine the amount of current needed at each frequency (5 Hz, C fibers; 250 Hz, Aδ fibers; 2000 Hz, Aβ fibers) to elicit a paw withdrawal. There was a small but significant decrease in the amount of current needed at 2000 Hz to elicit a paw withdrawal at weeks 1 and 2 of treatment, which resolved back to baseline in weeks 3-4 (A). Bortezomib induced a significant decrease in the amount of current needed at 250 Hz to elicit a paw withdrawal at one week, which persisted through week 4 (B). Bortezomib induced a significant decrease in the amount of current needed at 5 Hz to elicit a paw withdrawal at one week, which persisted through week 4 (C).

Sensory fiber function was assessed by determining the CPT in the hind paw using the Neurometer device, which delivers transcutaneous electrical stimuli at frequencies that have been previously reported to be specific to each fiber type (Aβ -2000 Hz; Aδ -250 Hz; C -5 Hz; Koga et al., 2005). Bortezomib treatment induced a significant and persistent decrease in the amount of current required to induce a paw withdrawal to the 5 Hz (Figure **6B**; p<0.01 vs. naïve and vehicle-treated mice) and 250 Hz (Figure **6D**; p<0.01 weeks 1 & 2; p<0.001 weeks 3 and 4 vs. naïve and vehicle-treated mice) stimulation frequencies. By contrast, there was only a small (although significant) difference in the amount of current required to elicit a paw withdrawal using the 2000 Hz stimulation between the drug and vehicle-treated groups (Figure **6F**; p<0.05 vs. naïve and vehicle-treated mice), which resolved back to baseline in the third week. These data suggest that the Aδ and C fibers are sensitized early after bortezomib treatment in hind paw, such that less current is required to stimulate a response.

#### 6: Assessment of morphological alterations: light microscopy

**Figure 7:**
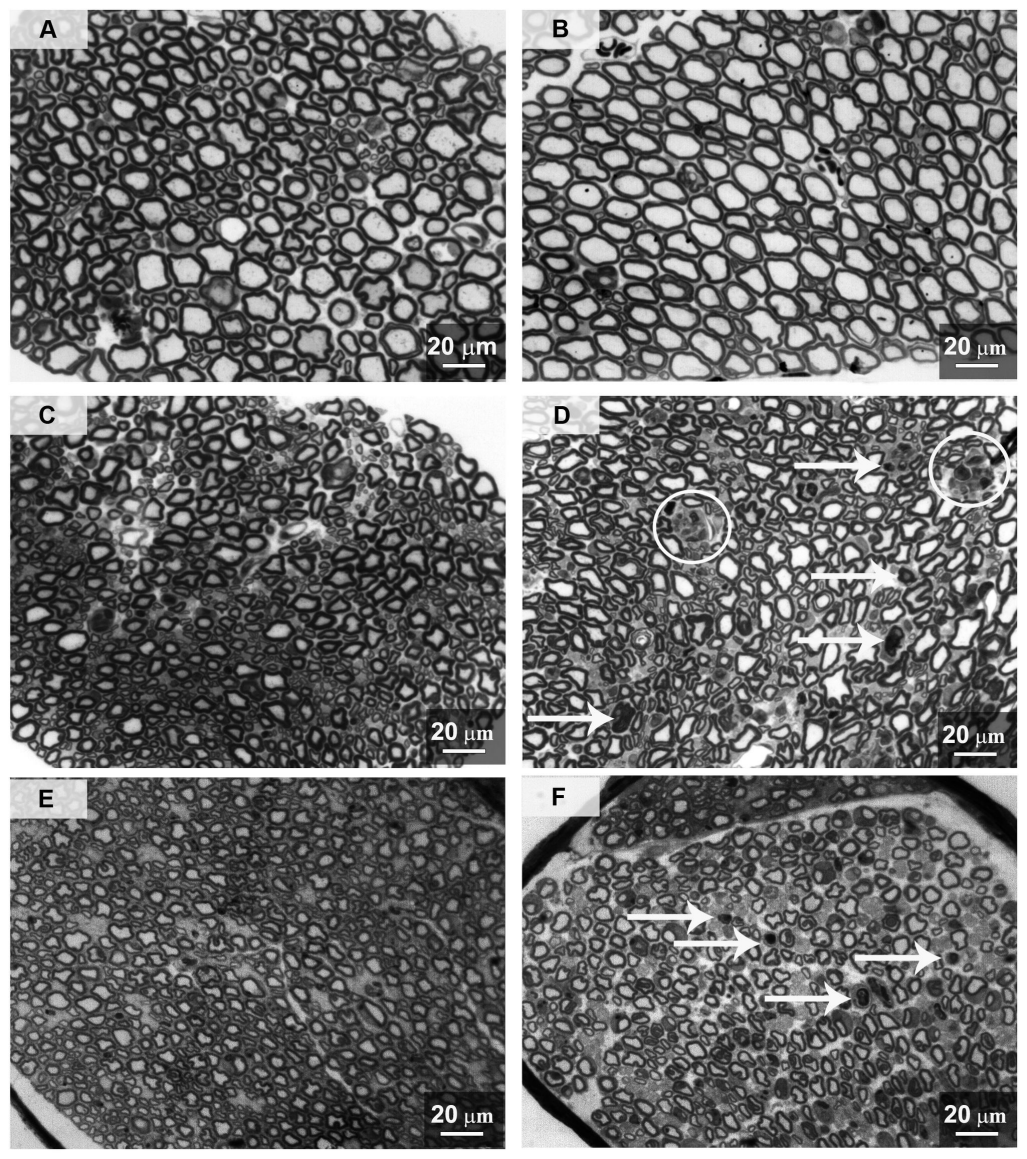
Morphological analysis of ventral/dorsal roots and caudal nerve, experiment 1. Dorsal and ventral roots and caudal nerves were collected at sacrifice and processed for light microscopy. A: vehicle ventral root, B: bortezomib ventral root, C: vehicle dorsal root, D: bortezomib dorsal root, E: vehicle caudal nerve, F: bortezomib caudal nerve. Bortezomib did not alter the morphology of the nerve fibers in the ventral roots (B). Bortezomib induced some morphological alterations in the axoplasm and myelin of the nerve fibers in dorsal roots (arrows in D), sometimes leading to advanced axonal degeneration (circles in D). Moderate to severe axonal degeneration of the myelinated and unmyelinated fibers was evident in the caudal nerves of bortezomib-treated animals (arrows in F). Ventral root, dorsal root and caudal nerve of vehicle-treated mice did not manifest any morphological alterations.

**Figure 8:**
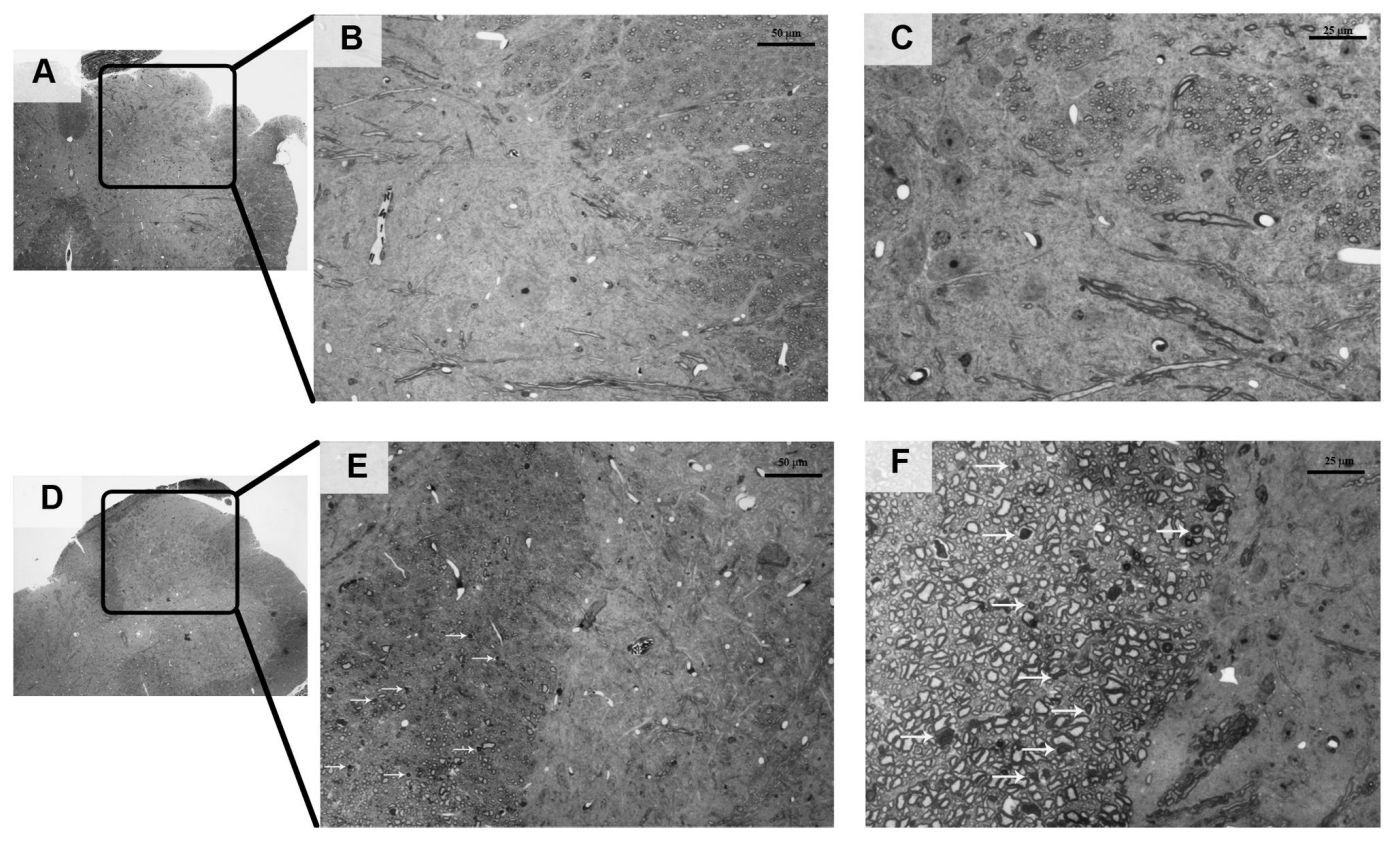
Morphological analysis of lumbar spinal cord, experiment 1. Lumbar spinal cords from vehicle- (A, B, C) and bortezomib-treated (D-E-F) mice were collected at the time of sacrifice and processed for light microscopy. No evident alterations of dorsal horn neurons were found while sporadic axonal degeneration was present in the myelinated fibers of the dorsal column of bortezomib-treated mice (arrows in E and F) compared to the vehicle-treated ones (B-C).

Morphological examination of the peripheral nerves, dorsal/ventral roots, DRG and lumbar spinal cord was done to determine whether any pathological changes were present. Three mice from each group were sacrificed after the last administration of bortezomib and used for the sample collection and processing. Thin sections of dorsal/ventral root and caudal nerve tissues were examined at the light microscope level (Figure **7 A-F**). These observations revealed no significant alterations in the morphology of the L4-L5 ventral roots of bortezomib-treated mice compared to the vehicle-treated mice (Figure **7 A, B**). By contrast, the dorsal roots of bortezomib-treated mice showed morphological alterations in the nerve fiber structure in both the axoplasm and the myelin sheaths compared to vehicle-treated mice (Figure **7 C, D**), sometimes leading to advanced axonal degeneration (circles in Figure **7 D**). As previously shown [21], bortezomib induced moderate to severe axonal degeneration of the myelinated and unmyelinated fibers in the sciatic nerves (data not shown). Similar, but more severe, alterations (Figure **7 E**) were also observed in the caudal nerve of bortezomib-treated compared to the vehicle-treated mice (Figure **7 F**). Dorsal horn neurons showed no evidence of morphological abnormalities while sporadic alterations (i.e. axonal degeneration) in the myelinated fibers of the dorsal column were observed (Figure **8 D, E, F**) compared to the vehicle-treated mice (Figure **8 A, B, C**). According to Carozzi et al. [21], DRGs of mice treated with bortezomib frequently showed damage of sensory neurons and satellite cells that was not observed in the vehicle-treated mice (data not shown). No differences between vehicle-treated and naïve mice were evident in this study.

#### 7: Qualitative assessment of DRG neuronal injury through ATF3 immunolabeling

**Figure 9:**
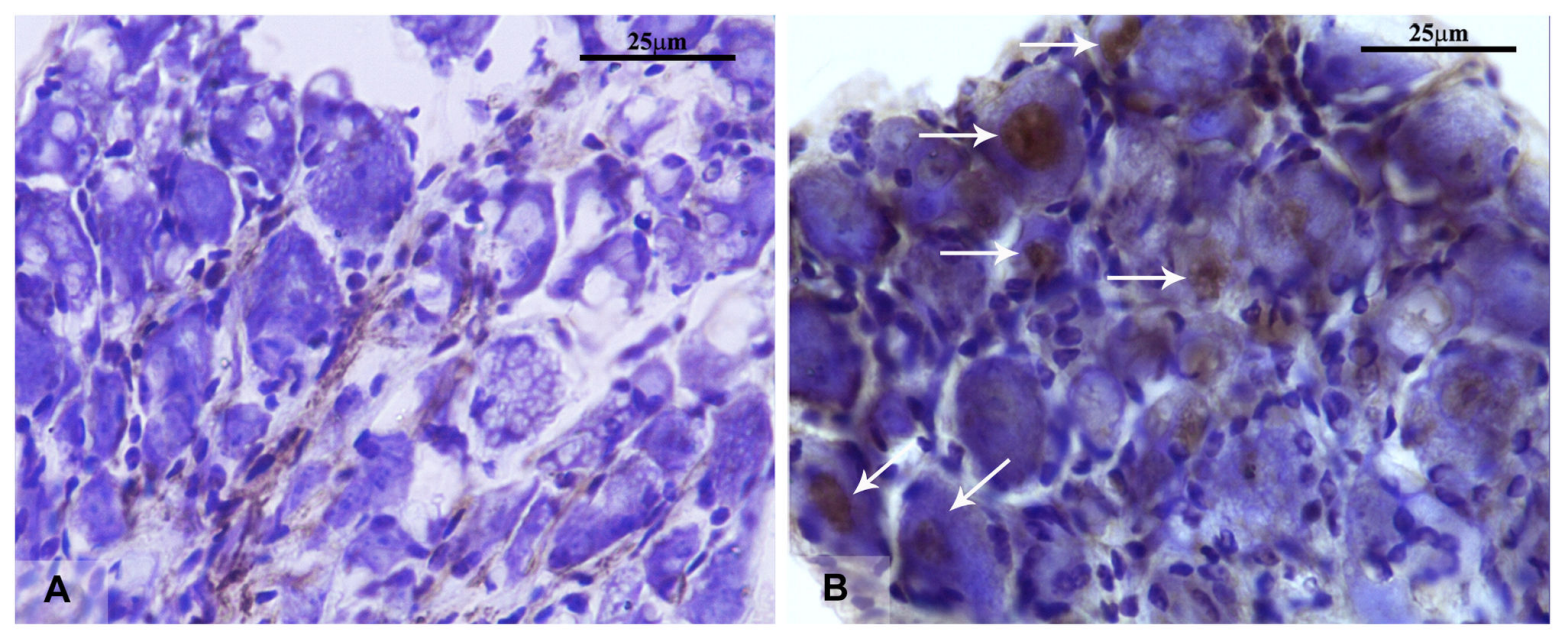
ATF3 immunolabeling, experiment 1. DRG were collected at the time of sacrifice for the immunohistochemical detection of the nuclear transcription factor ATF3. Thick (15 µm) cryostat sections of DRG were collected from vehicle (A) and bortezomib-treated (B) animals. Bortezomib treatment induced a marked nuclear ATF3 expression in DRG sensory neurons (arrows in B). No ATF3-positive nuclei were present in DRG of vehicle-treated mice.

Immunohistochemical analysis for the presence of ATF3 was performed on L4-L5 DRG harvested at the end of bortezomib treatment. ATF3, a transcription factor, is considered a standard marker of neuronal injury, even in the absence of evident pathological changes. As shown by white arrows in Figure **9B**, chronic treatment with bortezomib induced a marked upregulation of ATF3 in most DRG neuron nuclei. ATF3 was not detectable in the DRG of vehicle-treated animals (Figure **9A**). No differences between vehicle-treated and naïve mice were evident.

### Experiment 2

#### 1: General appearance and assessment of body weight changes

During the experimental period, mice were monitored daily for signs and symptoms of sickness and for general behavioral changes. During the 72 hours after the first X-Ray irradiation, most of the mice showed moderate signs of mild hypokinesia and piloerection, which resolved within one week. One mouse died after the second 100 RAD irradiation. The treatment with bortezomib, starting 24 hours after the last 100 RAD-irradiation, was fairly well tolerated by the mice. However, 50% of the mice showed kyphosis, piloerection, hypokinesia and pale skin.

**Figure 10:**
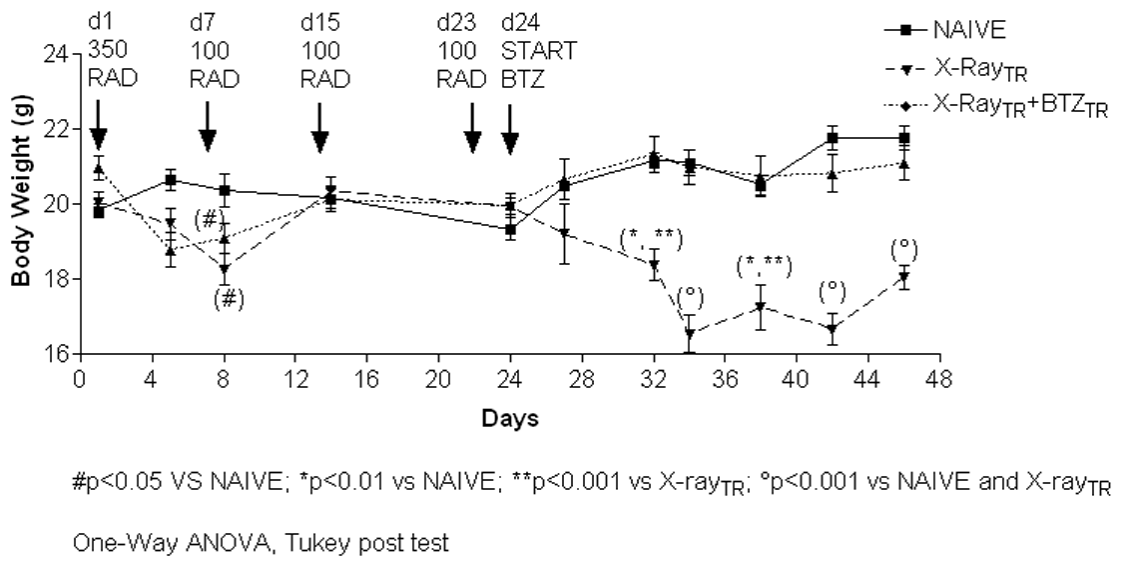
Body weight, experiment 2. Body weights were measured twice a week to monitor X-Ray irradiation and bortezomib-induced toxicity. X-Ray-treated animals showed an initial slight body weight decrease on day 4 and 8 then resumed a normal growth trend similar to naïve animals. Starting from the second bortezomib administration (day 32) animals showed a marked and statistically significant decrease of body weight that persisted till the end of the experimental period.

Body weight measures showed a significant decrease due to X-Ray irradiation one day after the first 100 RAD dose (day 8), which resolved by day 15 (Figure **10**, p<0.05 vs. naïve). After the beginning of bortezomib treatment, mice began to lose weight, which reached statistical significance after the third drug injection compared to naïve and X-Ray-treated mice (Figure **10**; day 32 and 38 p<0.01 vs. naïve and p<0.001 vs. X-Ray-treated; day 34 and 42 and 46, p<0.001 vs. naïve and X-Ray-treated). No mice died or needed premature euthanasia during the experiment.

#### 2: WBC count and flow cytometer analysis

Immune-suppression in the mice was assessed at 24 and 72 hours after the first X-Ray irradiation by evaluating the cellularity of the BM and PB and by flow cytometer analysis. The efficacy of the irradiation-induced immune-suppression was confirmed by the absolute WBC count and a decrease of the pan-leukocyte CD45 antigen expression in both the BM and the PB. By Flow Cytometer analysis we further observed a general depletion of other hematopoietic compartments, such as the Gr1+ myeloid, the CD3+ T-lymphoid, the CD19+ B-lymphoid, and the NK1.1+ natural killer cells (data not shown).

**Figure 11:**
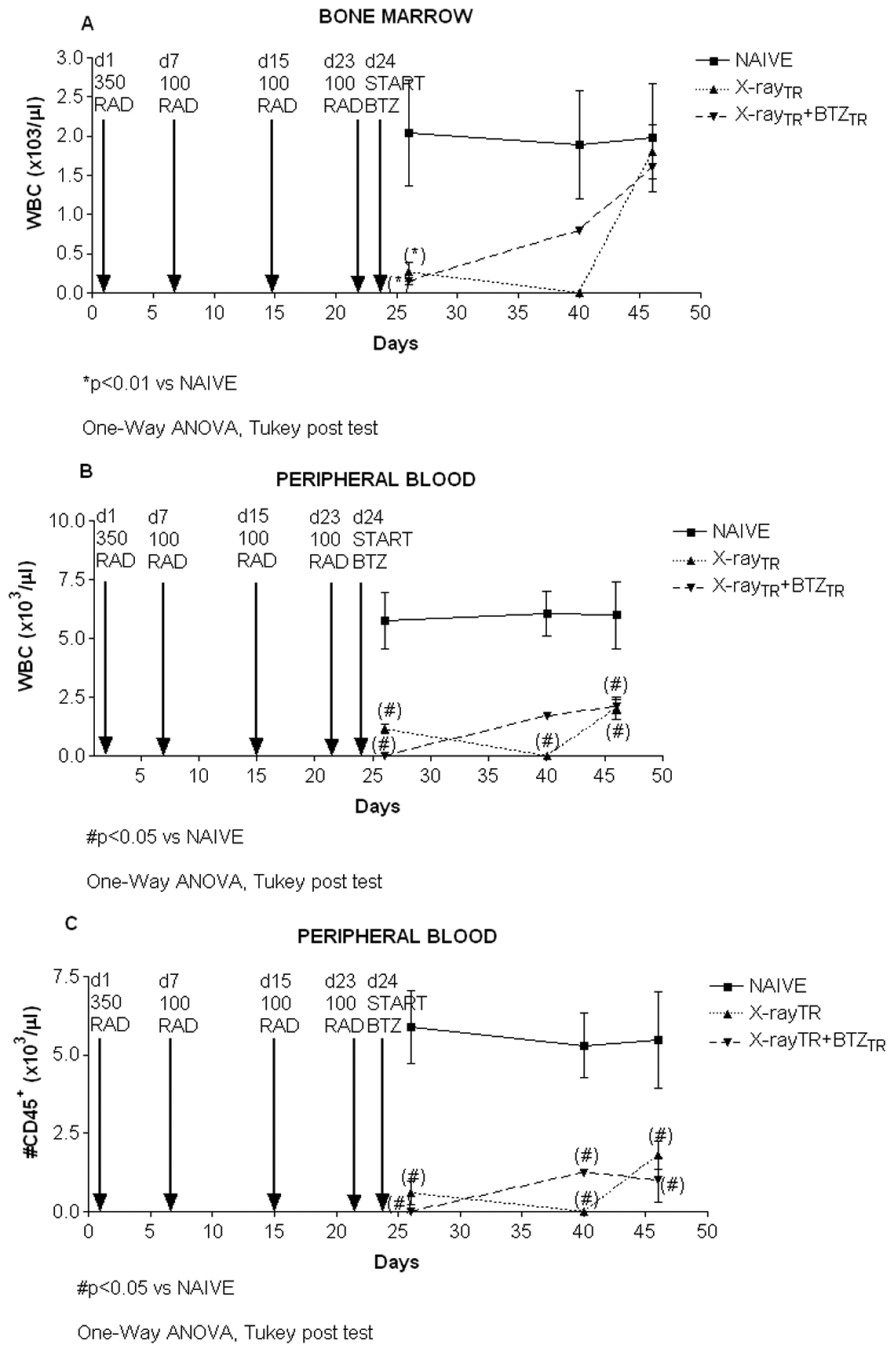
Bone marrow and peripheral blood and bone marrow white blood cells (WBC) count and FACS analysis, experiment 2. Black arrows indicate the treatment given to mice, such as the X-ray irradiation doses (350 RAD induction and 100 RAD maintenance), and bortezomib administrations. For each group of animals, the WBC concentration within the BM (A) or peripheral blood (B) is indicated for the corresponding analysis time point. The number of hematopoietic cells in the peripheral blood expressing the pan-leukocyte antigen CD45 is also reported (C). A comparable immune-suppression was observed in both X-Ray and X-Ray-bortezomib-treated animals after the irradiation induction and maintenance doses, as shown by the decreased cellularity and leukopenia in the BM and spleen of irradiated mice, regardless of bortezomib treatment.

After the third maintenance irradiation dose (100 RAD), the WBC depletion was still statistically significant in BM and PB of both X-Ray and X-Ray-BTZ groups of mice compared to controls (Figure **11A & B** respectively). Similarly, CD45+ leukocytes were completely absent in the PB of irradiated mice as a consequence of X-Ray treatment, regardless of BTZ treatment (Figure **11C**). The immune-suppression persisted in the PB throughout the experimental period, as assessed by periodic time point analysis (Figure **11B & C**). However, at the final analysis time point, we observed a partial recovery of the hematopoietic cells, but only within the BM compartment (Figure **11A**), mainly arising from the B-lymphocyte cells compartment (data not shown).

#### 3: Neurophysiological assessments in the peripheral nerves

**Figure 12:**
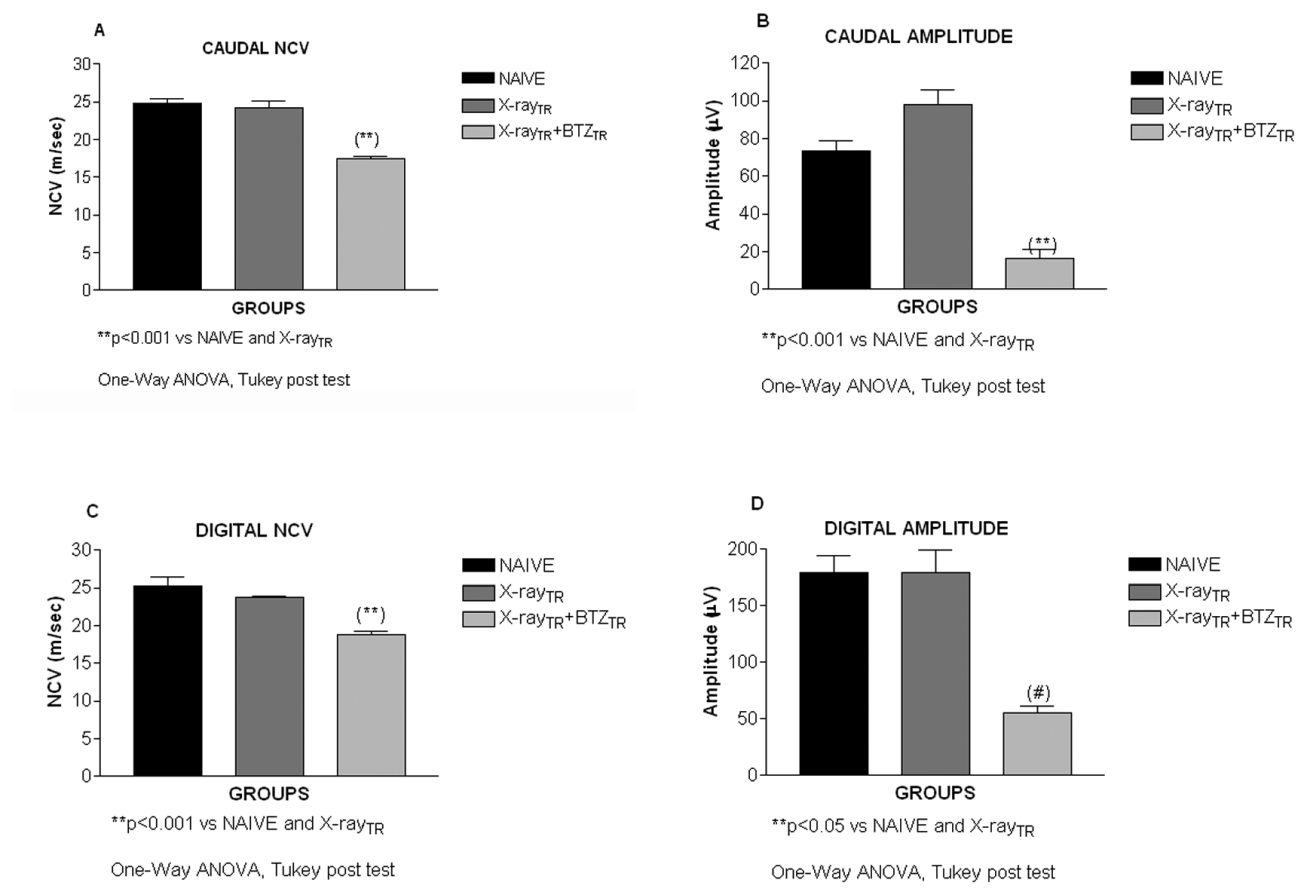
Neurophysiology, experiment 2. Nerve Conduction Velocity (NCV) and action potential amplitudes were tested with an electromyographic instrument in the caudal and digital nerves two days after last bortezomib administration. X-Ray irradiation alone did not alter the NCV while bortezomib induced a significant decrease of the caudal (A) and digital (C) NCV compared to naïve and X-Ray animals. X-Ray irradiation alone did not alter the action potential amplitude while bortezomib induced a statistically significant reduction of the caudal (B) and digital (D) action potential amplitude compared to naïve and X-Ray animals.

As described above for **experiment 1**, recordings of the NCV and neuronal action potential amplitude were done at the end of the 4-week period of bortezomib treatment. The X-Ray irradiation alone did not induce any neurophysiological change (Figure **12**; p>0.05 for X-Ray treated mice compared to naïve) in either the caudal or digital nerves. By contrast, the 4-week period of bortezomib treatment after X-Ray irradiation induced a significant reduction of the caudal and digital NCV and action potential amplitude (Figure **12A & B**; p<0.001 vs. naïve and X-Ray-treated mice; Figure **12C**; p<0.001 vs. naïve and X-Ray-treated mice; Figure **12D**; p<0.05 vs. naïve and X-Ray-treated mice).

#### 4: Assessments of mechanical thresholds

**Figure 13:**
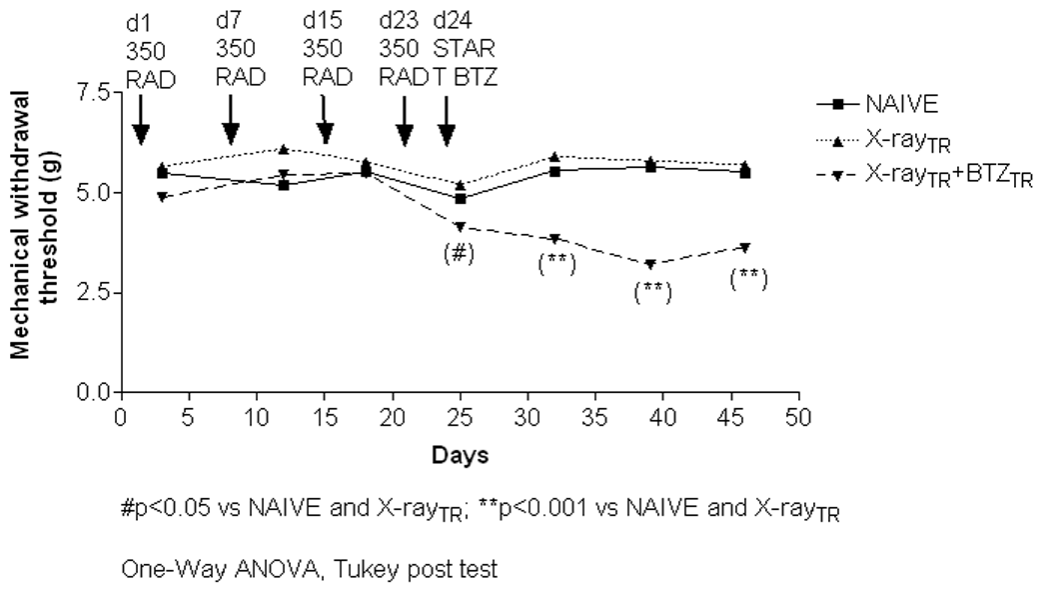
Mechanical threshold, experiment 2. The mice were tested weekly using the Dynamic Aesthesiometer instrument. X-Ray alone did not induce any alteration in the withdrawal threshold to mechanical stimulation while bortezomib induced a significant decrease in the mechanical threshold starting from one day after the first injection (day 25) and persisting through the end of the pharmacological treatment.

To determine if the X-Ray irradiation alone or combined with bortezomib treatment was able to induce the development of neuropathic pain, mice underwent the weekly assessment of the nocifensive response to mechanical stimulation using the Dynamic Aesthesiometer. No significant alterations in the response to mechanical stimulation were evident in the X-Ray-irradiated compared to naïve mice (Figure **13**). By contrast, mice that received bortezomib after the X-Ray irradiation had a significant decrease in the mechanical threshold starting 1 day after the beginning of bortezomib treatment (day 25) and persisting until the end of the experiment (Figure **13**; day 25 p<0.05 vs. naïve and X-Ray-treated mice; days-32-39-46; p<0.001 vs. naïve and X-Ray-treated mice).

#### 5: Assessment of morphological alterations: light microscopy analysis

**Figure 14:**
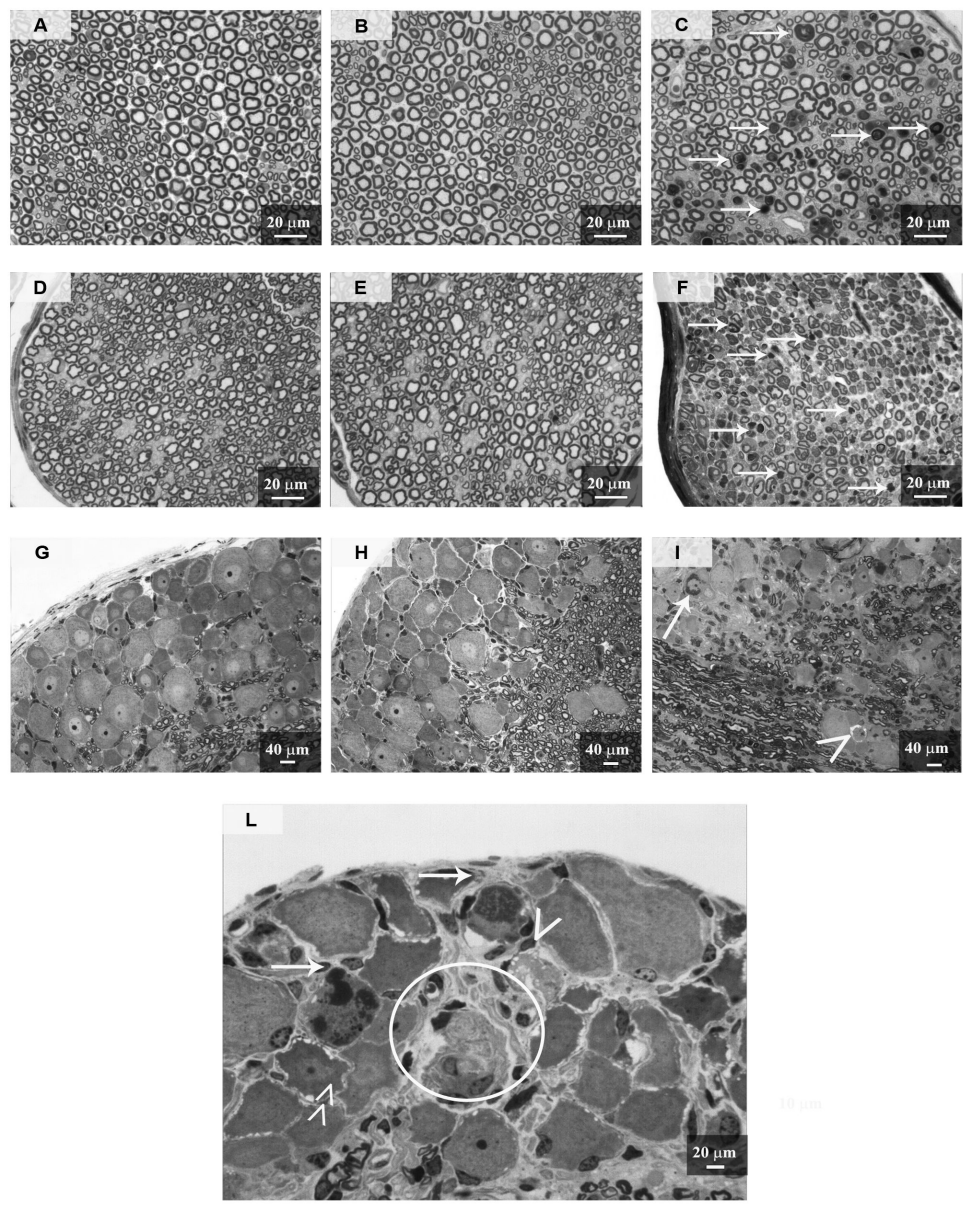
Morphological analysis of sciatic and caudal nerves and DRG, experiment 2. DRG, sciatic and caudal nerves were collected at sacrifice and processed for light microscopy. A: naïve sciatic nerve, B: X-Ray sciatic nerve, C: X-Ray + bortezomib sciatic nerve, D: naïve caudal nerve, E: X-Ray caudal nerve, F: X-Ray + bortezomib caudal nerve, G: naïve DRG, H: X-Ray DRG, I, L: X-Ray + bortezomib DRG. No morphological alterations were evident in naïve and X-Ray-treated mice. Bortezomib induced moderate to severe morphological alterations in the axoplasm and myelin of the nerve fibers in sciatic (C) and caudal (D) nerves (arrows). Bortezomib induced the formation of dark inclusions and clear vacuolization in the cytoplasm of sensory neurons (arrows and arrowheads respectively in L), leading in some cases to neuronal degeneration (circle in L). Bortezomib determined sporadic vacuolization of cytoplasm in satellite cells (double arrowhead in L). DRG=Dorsal Root Ganglia.

Sections of sciatic nerve, caudal nerve and DRG were examined for X-Ray and bortezomib-induced morphological changes. X-Ray alone did not induce any morphological alteration (Figure **14 B, E, H**) compared to the naïve mice (Figure **14 A, D, G**). By contrast, the sciatic nerves of X-Ray and bortezomib-treated mice showed morphological abnormalities of the myelinated fibers in different stages of severity (Figure **14 C**), with evidence of myelin and axoplasm deterioration leading to the fiber collapse with the aspect of Wallerian-like degeneration. Similar, but more severe changes were also present in caudal nerves (Figure **14 F**). The DRGs were also affected by X-Ray treatment followed by bortezomib administration, which caused morphological alterations in the sensory neurons (i.e. dark inclusions and clear vacuolizations throughout the cytoplasm; arrows and arrowheads, respectively, in Figure **14 I, L**. Sometimes, DRG neuronal degeneration was observed (circle in Figure **14L**) and sporadic cytoplasmic vacuolizations were also evident in satellite cells (double arrowheads in Figure **14 L**).

## Discussion

Bortezomib is the standard drug in the chemotherapy regimen used to treat MM [2,3,38]. Unfortunately, despite it is a highly effective anticancer drug, it causes a dose-limiting painful PN in up to 30% of patients when given intravenously [9,12,39,40,41]. Although neuropathic pain is by far the most worrisome side effect of bortezomib therapy, there is also clinical evidence that the drug induces different degrees of impairment in the functioning of Aβ, Aδ and C fibers [42]. Since bortezomib is likely to remain the standard of care for MM treatment also in the near future, recent studies help to increase our understanding of bortezomib-induced PN. However, the real extent and sites of nervous system damage as well as the underlying mechanisms responsible for its onset remain unclear and require therefore further investigation. In this study, we first expanded previous observations on the effects of bortezomib treatment on the function and integrity of the peripheral nervous system by including the investigation also of the caudal nerve, spinal roots and spinal cord and then we investigated the role of the immune response in the development and severity of bortezomib painful PN in the mouse.

We demonstrated that bortezomib treatment-induced changes are not limited to the DRG, sciatic as it was previously demonstrated by Carozzi et al. [21]. In fact, it also causes evident pathological changes in the caudal nerve dorsal roots and dorsal columns of the spinal cord. Functional abnormalities of WDR dorsal horn spinal cord neurons were also observed. Neurophysiological abnormalities, evidenced by a decrease of the caudal and digital nerve conduction velocities and potential amplitudes, were almost similar to previous experimental studies in rats [19,21,43] and to clinical findings where sensory and motor amplitudes (and to a lesser extent, conduction velocities) were reduced in more than 70% and 30% of patients, respectively [10,11,44]. Since NCV studies inform principally about the changes occurring in the largest (Aβ) fibers, we searched for selective functional effects of bortezomib in our mouse model on the three sensory fiber types using the CPT test [45]. This test has already been used to assess the Aβ and Aδ fibers functionality of diabetic, paclitaxel-treated and sciatic nerve injured animals [35,46,47]. In this work, the Aβ fibers showed a limited, although statistically significant sensitization in the first two weeks of bortezomib treatment that subsequently resolved. These results are not in disagreement but rather complementary to the NCV studies, since CPT assessment explores the entire sensory pathway while NCV reliably measures only the effects on the peripheral nerves. Also the nociceptive Aδ and C-fibers became sensitized early after the onset of bortezomib treatment, but this effect persisted through the end of the study. The observed behavioral changes, represented by early onset of mechanical allodynia without heat and hyperalgesia, are consistent with the results of previous experimental studies with bortezomib [19,21,22] and they partially mimic the clinical experience where also cold detection thresholds are altered [12,42]. Our results suggest a differential damage involving mechanosensitive rather than thermosensitive fibers which is different from the neurotoxic effects of other chemotherapy agents that induce similar mechanical and thermal hypersensitivity. As an example, chronic treatment with the mitotic inhibitor vincristine induced mechanical hyperalgesia and allodynia associated with cold thermal hyperalgesia and allodynia in rats [48].

As we previously reported [21], bortezomib treatment is associated with the development of axonopathy in the peripheral nerves and pathological changes of DRG neurons and satellite cells. However, here we demonstrated for the first time that bortezomib treatment also induces degeneration of the myelinated fibers in the dorsal roots and in the dorsal columns of the spinal cord. Since bortezomib is unable to cross the blood brain barrier, it is likely that these changes are secondary to DRG neuronopathy. This observation is intriguing, in view of the pathological report of a patient affected by MM and treated with bortezomib where degeneration of the dorsal column from the cervical spinal cord to the medulla oblongata was found [49]. In an attempt to correlate our pathological findings with the behavioral results it should be considered that in rats and mice tactile perception and nociceptive signals arising from the periphery are transmitted to supra-spinal sites through the dorsal column-medial lemniscal and anterolateral systems, respectively [50,51]. As a matter of fact, the dorsal column-medial lemniscal system has been considered to be an important ascending pathway for mechanical allodynia [50]. As explained by Cervero and Laird [16], the excitation of nociceptors provoked by a peripheral injury activates the spinal interneurones that mediate primary afferent depolarization between low threshold mechanoreceptors and nociceptors. The excitability of these neurones is increased and when activated by low threshold mechanoreceptors from the injury site, they produce a very intense primary afferent depolarization in the nociceptive afferents which is capable of generating spike activity. The sensory consequence of this mechanism is allodynia, a pain evoked by the activation of low threshold mechanoreceptors from the injury site.

To support our findings, we also characterized bortezomib-induced damage in DRG neurons through immunohistochemical detection of ATF3, a neuronal injury marker that is highly expressed in several models of neuropathic pain even in absence of gross changes in DRG neurons morphology [52,53,54]. Using this staining we demonstrated that bortezomib induced an evident expression of ATF3 in the nuclei of most DRG sensory neurons, but not of the spinal cord neurons. The events that induced ATF3 overexpression in the DRG neurons likely involved some degree of axonal injury, which signals the cell body to upregulate ATF3 expression [55]. ATF3 overexpression in sensory neurons has also been shown in experimental models of cisplatin, paclitaxel and diabetes-induced PN [56,57,58]. The co-expression of c-jun and JNK with ATF3 in the nervous system following traumatic injury and other stressful stimuli suggests that upregulation of c-jun and JNK may be followed by activation of ATF3. This is in agreement with data showing that bortezomib-induced proteasome inhibition increased phospho-c-jun-NH(2) and JNK kinase in cultured melanoma cells [59] and DRG primary sensory neurons [60]. Moreover, it is conceivable that ATF3 upregulation is present in sensory neurons with axons projecting into damaged peripheral nerves [53]. Moreover, it was also demonstrated that ATF3 expression in the nucleus of injured neurons is closely correlated with a regenerative response [54,61]. In fact, since in transgenic mice that constitutively express ATF3 in non-injured adult DRG neurons, peripheral nerve regeneration is enhanced [62], ATF3 contributes to nerve regeneration by increasing the intrinsic growth state of injured neurons [54]. Regenerative capacity of DRG neurons of mice exposed to chronic bortezomib treatment has to be elucidated.

Given that the bortezomib-treated mice developed neurophysiological, morphological and molecular changes from the peripheral nerves to the dorsal column of spinal cord, we then investigated the electrical activity of WDR neurons in the lumbar enlargement of the spinal dorsal horn, since a variety of neuropathic pain models exhibit increased evoked WDR neuron activity after nerve injury [63,64,65,66]. Here we found that WDR neuron activity in response to innocuous, moderate and noxious hind paw stimulation was significantly increased, in complete agreement with the observed development of mechanical allodynia. The increased activity of the WDR neurons in our model might be due to increased neurotransmitter release in the spinal dorsal horn and/or to a decreased frequency of inhibitory synaptic events [67,68], leading to altered synaptic transmission and exaggerated excitatory activity [69,70]. Therefore, our results, which are similar to those obtained in other chemotherapy-induced neuropathy models [33], may reflect physiological changes in the spinal dorsal horn as well as in primary afferent fibers [33,64,71,72,73,74].

In the second part of the study, we explored the effect of bortezomib administration in immune-deficient mice to test the hypothesis that the immune response might be relevant to the development and/or severity of bortezomib-induced PN. The main reason for this pathogenetic investigation is that nerve biopsies of neuropathic patients that underwent bortezomib therapy showed an increase in the reactivity for the CD3 surface antigen and for the antigen CD68, as well as the presence of perivascular activated T cells and endoneural macrophages [75]. These cells can release pro-inflammatory cytokines (Tumor Necrosis Factor α, IL-1beta, interleukins) that can sensitize primary sensory afferents and modify afferent input to the spinal dorsal horn to facilitate pain. On this basis, immune modulation has been proposed as a possible treatment for bortezomib-induced PN [30]. Moreover, in a recent clinical study, a cohort of MM patients that received a bortezomib-based chemotherapy regimen was tested for their neurological, neurophysiological and inflammatory status (CD4, CD8 T cell subpopulations, CD4+ sub-populations, pro-inflammatory interleukins profile). Some of the patients with neuropathic pain and neurophysiological abnormalities showed significant modifications in Th1 and Th2 cell subsets and a concomitant increase of IL-6 levels [76]. To address this issue in BALB/c mice, we developed **a** new *ad hoc* immunodeficient mouse model based on X-Ray irradiation at sub-lethal doses. This type of X-Ray exposure results in pancytopenia and, eventually, in a complete inhibition of innate and acquired immunity, antibody production and cell-mediated response [77]. After irradiation, the immunodeficient animals were exposed to the same 4 week-treatment with bortezomib used in the first part of the study. Behavioral tests, neurophysiological analysis in peripheral nerves and morphological observation of sciatic and caudal nerves and DRG were performed and demonstrated that the immunodeficient animals treated with bortezomib developed a painful PN with the same features observed in the immunocompetent mice, thus making it very unlikely that the immune response is a key factor in the pathogenesis of the severe damage induced by bortezomib in our experimental paradigm.

## Conclusions

Our study demonstrates that the neurotoxic effect of chronic bortezomib treatment in mice is not limited to the PNS, but also extends to the CNS. Given the inability of bortezomib to cross the blood-brain barrier, it is conceivable that the observed changes in the spinal cord are secondary to DRG neuron and peripheral nerve fibers damage that was much more extensive than expected based on the simple morphological examination previously reported. Finally, the pathogenetic role of an immune response in the onset of bortezomib-induced damage is challenged by the results observed in the severely immunodeficient mice used in our study.
